# STAGE framework: A stock dynamic anomaly detection and trend prediction model based on graph attention network and sparse spatiotemporal convolutional network

**DOI:** 10.1371/journal.pone.0318939

**Published:** 2025-03-17

**Authors:** Ming Shi, Roznim Mohamad Rasli, Shir Li Wang

**Affiliations:** 1 Faculty of Computing and Meta-Technology, Universiti Pendidikan Sultan Idris, Tanjong Malim, Perak, Malaysia; Ningbo Institute of Digital Twin, Eastern Institute of Technology, CHINA

## Abstract

As the financial market becomes increasingly complex, stock prediction and anomaly data detection have emerged as crucial tasks in financial risk management. However, existing methods exhibit significant limitations in handling the intricate relationships between stocks and addressing anomalous data. This paper proposes the STAGE framework, which integrates the Graph Attention Network (GAT), Variational Autoencoder (VAE), and Sparse Spatiotemporal Convolutional Network (STCN), to enhance the accuracy of stock prediction and the robustness of anomaly data detection. Experimental results show that the complete STAGE framework achieved an accuracy of 85% after 20 training epochs, which is 10% to 20% higher than models with key algorithms removed. In the anomaly detection task, the STAGE framework further improved the accuracy to 95%, demonstrating fast convergence and stability. This framework offers an innovative solution for stock prediction, adapting to the complex dynamics of real-world markets.

## 1 Introduction

### 1.1 Background

In the complex environment of modern financial markets, stock prediction has become a widely studied research field. Market dynamics and investment decisions face unprecedented challenges and opportunities. Stock prices are influenced by a multitude of factors, including macroeconomic environment, market sentiment, and socio-economic dynamics. With the advancement of related research, more people recognize that stock market volatility is greatly influenced by social media and public sentiment. For instance, in August 2018, Elon Musk’s tweet about taking Tesla private led to a 7% rise in the stock price, while another tweet stating that the price was “too high” caused a 10% drop. In January 2021, the Reddit user-led short squeeze on GameStop and AMC Entertainment drove their stock prices up to $400 (an increase of over 1900%) and $73 (an increase of 3500%), respectively, seriously affecting market stability. MARYLAND SMITH RESEARCH has also shown that a company’s tweet sentiment can trigger short-term fluctuations in its stock price.

With the rapid development of information technology, researchers have gradually shifted from traditional statistical analysis to more advanced machine learning and deep learning techniques [12]. However, existing prediction models face two main challenges when dealing with complex stock market data [12,13]. In terms of stock relationship modeling, existing methods have difficulty effectively capturing the complex nonlinear correlation patterns between stocks. Especially during periods of severe market fluctuations, traditional models fail to accurately characterize the dynamic mutual influence between stocks, resulting in poor predictive performance [14]. Additionally, in terms of anomaly detection, existing models have limited capabilities for integrating multi-source data, making it difficult to identify and respond to abnormal market fluctuations in a timely manner. Such prediction inaccuracies during critical moments not only affect model practicality but also introduce significant uncertainty and risk into investment decisions.

### 1.2 Literature review

In the current field of stock prediction research, an increasing number of researchers have started using hybrid models to improve prediction accuracy and stability. Xu et al. proposed an enhanced nonlinear fusion model based on GAN, integrating ACNN, LSTM, and ARIMA models for effective stock price prediction, demonstrating its superiority in experiments [2]. However, the model still has limitations in capturing complex temporal features. Conversely, Dong et al. combined the SARIMA model with the Monte Carlo method to overcome the limitations of single models, proposing a hybrid approach for stock value prediction to enhance accuracy [3], but it lacked consideration for external variables such as macroeconomics, limiting the model’s flexibility. Meanwhile, the application of deep learning methods in the stock prediction domain has also been on the rise. Liu et al. introduced a deep learning model that integrates mixed-frequency data for stock volatility prediction, showing good performance in handling high-frequency data [5], though there remains room for improvement in capturing high-frequency data features.

To address prediction challenges in complex market environments, Zhang et al. proposed the MDF-DMC model, which combines multi-perspective stock data features and dynamic market-related information, effectively enhancing the accuracy of stock price prediction by dynamically learning correlations between stocks [10]. However, the model’s stability under complex market conditions still requires further verification. Wang et al. proposed a model based on PCA and improved IGRU, focusing on reducing redundant input information to improve model training efficiency and prediction performance [4], but the method still lacks in considering the interrelationships between stocks.

Current research trends mainly focus on using hybrid models, deep learning methods, and dynamic fusion of data features to overcome the shortcomings of traditional methods in terms of prediction accuracy and capturing market dynamics [27]. However, these methods still face challenges in the comprehensiveness of data features, integration of external factors, and model adaptability and stability, necessitating further research and improvement, as shown in Table 1. The application and improvement of these methods provide new ideas and stronger interpret ability for stock prediction, but to fully utilize their potential in practical applications, the existing limitations must be addressed.

**Table 1:** Literature review table.

Author	Application Scenario	Research Content	Potential Limitations
Zheng, Xiuyan, et al. [1]	Stock Trend Prediction	Proposed a hybrid model based on ARIMA-LightGBM for stock trend prediction, combining ARIMA and LightGBM models to predict the future trend of Gree Electric’s stock	The model is mainly suitable for single data sources, lacking applicability to multi-source complex relationships
Xu, Yingcheng, et al. [2]	Stock Price Prediction	Proposed an enhanced nonlinear fusion model based on GAN, integrating ACNN, LSTM, and ARIMA models for effective stock price prediction	The ability to capture complex temporal features still needs improvement
Dong, Chunyao, et al. [3]	Stock Value Prediction	Proposed a hybrid model combining the SARIMA model and Monte Carlo method for stock value prediction	Lacks consideration of external variables such as macroeconomics, leading to limited model flexibility
Wang, Jingyang, et al. [4]	Stock Price Prediction	Proposed a model based on PCA and improved IGRU to reduce redundant input information and improve prediction accuracy	The method does not adequately consider the interrelationships and complexities between stocks
Liu, Wenting, et al. [5]	Stock Volatility Prediction	Proposed a method based on the Transformer model combined with mixed-frequency data for predicting stock volatility	There is still room for improvement in capturing high-frequency data features
Lv, Pin, et al. [6]	Stock Index Prediction	Proposed a hybrid model based on CEEMDAN-DAE-LSTM for stock index prediction, improving prediction accuracy through feature decomposition and denoising	The complexity of the hybrid model increases training time and implementation difficulty
Zhang, Qiuyue, et al. [7]	Stock Volatility Prediction	Proposed a fusion model based on collaborative attention Transformer, performing parallel feature extraction of text and stock prices for stock volatility prediction	Lacks integration of more types of textual data (e.g., social media), limiting model generalization
Muhammad, Tashreef, et al. [8]	Stock Price Prediction	Introduced the Transformer model to predict stock prices on the Dhaka Stock Exchange, applying time2vec encoding to stock time series prediction for the first time	Feature extraction is still limited by the periodicity of the data, and model adaptability needs enhancement
Zhang, Jilin, et al. [9]	Stock Price Prediction	Proposed a hybrid model based on CNN-BiLSTM-Attention to improve stock price prediction accuracy	Lacks analysis of feature importance, which may affect model interpretability
Yang, Zhen, et al. [10]	Stock Prediction	Proposed the MDF-DMC model, combining multi-perspective stock data features and dynamic market-related information to improve stock prediction accuracy	Model stability under complex market conditions requires further validation
Agrawal, Manish, et al. [11]	Stock Trend Prediction	Proposed a stock prediction model based on a deep learning model, using an evolutionary deep learning framework for more effective trend prediction	The ability to handle non-stationary data requires further validation

### 1.3 Our contributions

- **Combination of graph attention network and variational graph autoencoder:** This study proposes an innovative algorithm combining the Graph Attention Network (GAT) and Variational Graph Autoencoder (VGAE). GAT is used to efficiently aggregate correlations between stocks, while VGAE deeply encodes stock features in latent space to generate more representative high-dimensional latent space representations, capturing complex interactions between stocks more effectively.
- **Dynamic modeling with sparse spatiotemporal convolutional network:** To address the issue of anomalies in financial data, this study introduces the Sparse Spatiotemporal Convolutional Network (STCN). By dynamically modeling stock features in temporal and spatial dimensions, STCN can efficiently detect abnormal changes in the data and enhance sensitivity and robustness to complex dynamic features through sparse regularization.
- **Comparative ablation experiment on real dataset:** We conducted comprehensive comparative and ablation experiments on a real financial dataset to validate the effectiveness of the proposed STAGE framework in capturing complex relationships between stocks and addressing anomalies. The results show that the complete STAGE framework significantly outperforms simplified models with key modules removed in terms of prediction accuracy and robustness to anomalous data.

## 2 Methodology

### 2.1 Problem description

In the stock prediction problem, given a series of stock price data at different time points, the goal is to predict the stock price at a future time point. Suppose the time series data is $\left\{x_{1},x_{2},\ldots ,x_{t}\right\}$, our goal is to predict the stock price $x_{t+h}$ at time *ρ*.

Let the input sequence be $X=[x_{1},x_{2},\ldots ,x_{t}]$, and the corresponding predicted value be ${\hat{x}}_{t+h}$. The prediction process can be defined as a function *f* ( ⋅ ) , such that:


$$\begin{array}{c} {\hat{x}}_{t+h}=f(X)+ɛ(X), \end{array}$$ (1)

where *f* ( ⋅ )  represents the mapping process of the input sequence *X* to obtain the predicted value for the future time point, and  −  is the noise term, representing uncertainty and random disturbance in the prediction process. To improve the prediction performance, a composite loss function is often used to evaluate the error between the predicted value and the true value:


$$\begin{array}{c} L=\frac{1}{N}\overset{N}{\underset{i=1}{\sum }}\left(\alpha {\left({\hat{x}}_{t+h}^{\left(i\right)}-x_{t+h}^{\left(i\right)}\right)}^{2}+(1-\alpha )\left|{\hat{x}}_{t+h}^{\left(i\right)}-x_{t+h}^{\left(i\right)}\right|\right), \end{array}$$ (2)

where *N* represents the number of samples, ${\hat{x}}_{t+h}^{\left(i\right)}$ is the predicted value for the *i*-th sample, $x_{t+h}^{\left(i\right)}$ is the corresponding true value, and *α* is the balancing parameter that controls the weight between Mean Squared Error (MSE) and Mean Absolute Error (MAE).

To describe the anomaly data detection problem, suppose there are anomaly data points in the dataset *X*. Our goal is to detect these anomalies by minimizing the following objective function:


$$\begin{array}{c} J(\psi )=\frac{1}{M}\overset{M}{\underset{j=1}{\sum }}\ell (x_{j},{\hat{x}}_{j})+\beta \overset{K}{\underset{k=1}{\sum }}R_{k}(\psi ), \end{array}$$ (3)

where $\ell (x_{j},{\hat{x}}_{j})$ represents the loss function between the predicted value and the true value, *β* is the regularization parameter, $R_{k}(\psi )$ represents the *k*-th regularization term to enhance the model’s capability of detecting anomalies, and *K* is the number of regularization terms.

**Problem 1.** *The ultimate goal of stock price prediction can be expressed as:*


ψ∗= arg ⁡ min ⁡ ψ (1M∑j=1Mℓ(h(Yj;ψ),zj)+γ∑k=1Kgk(ψ)), (4)

*where $h(Y_{j};\psi )$ represents the mapping process of input data features,  ∗  is the loss function,  =  is the multi-objective weighting coefficient, $g_{k}(\psi )$ is the multi-dimensional socio-economic constraint function, and *K* is the number of constraint terms, which comprehensively considers the complex influence of various socio-economic factors on stock prices.*

### 2.2 Interaction between stocks: combination of graph attention network and variational graph autoencoder

#### 2.2.1 Synergistic advantages of graph networks and variational inference

- Traditional stock prediction methods often face limitations when capturing complex interrelationships between stocks, failing to effectively model dynamic associations and nonlinear features among stocks [13,15,26]. These methods typically rely on fixed statistical models or single neural network architectures, making it difficult to fully reflect the potential interactions between different stocks, thereby compromising the accuracy and reliability of prediction results [16,28].
- The proposed algorithm in this study combines the Graph Attention Network (GAT) with the Variational Autoencoder (VAE). Through graph structure modeling and latent space feature learning, it can effectively capture complex nonlinear relationships and dynamic interaction features among stocks. GAT is used to aggregate correlations between stocks, enabling the model to identify the influence of each stock in the overall market, while VAE further encodes stock features deeply to generate high-dimensional latent space representations, thereby enhancing the model’s robustness to anomalies and complex relationships.

**Fig 1:**
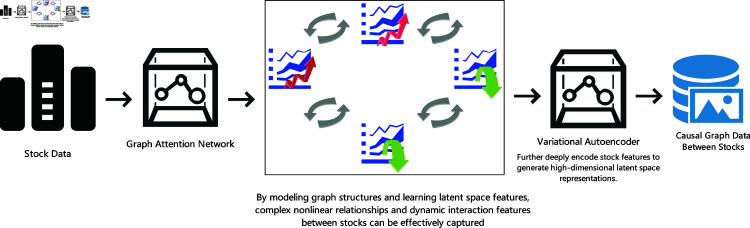
Capturing dynamic associations between stocks.

Fig 1 shows a framework of the stock prediction model. The framework inputs stock data into the Graph Attention Network (GAT) to learn complex interrelationships between stocks. Subsequently, the model uses the Variational Autoencoder (VAE) to deeply encode stock features and generate high-dimensional latent space representations.

**Algorithm 1: Stock relationship capture algorithm based on graph attentionand variational autoencoder.**

1:   

**Require:**   Stock set *V*, edge set *E*, adjacency matrix *A*, node feature matrix *X*

**Ensure:**   Final representation matrix of stock nodes *Z*

2:   **Initialize model parameters:**

3:   Initialize GAT parameters: weight matrix *W*, attention vector *a*

4:   Initialize VAE parameters: encoders $f_{\mu }$, $f_{\sigma }$, decoder $f_{\text{decoder}}$

5:   Initialize hyperparameters:  = , *p* < 0 . 1,  = 

6:   **Graph attention feature aggregation:**

7:   **for** each node  =  **do**

8:    Calculate attention weight $\alpha_{ij}$ between node *i* and its neighbor nodes *p* < 0 . 1 (refer to Eq 6)

9:    Update node feature $h_{i}^{\prime }$ (refer to Eq 5)

10:   **end** **for**

11:   **Variational autoencoder processing:**

12:   **for** each node  =  **do**

13:    **Encoding process:**

14:    Calculate the mean $\mu_{i}$ and variance log ⁡ σi2 of the latent variable (refer to Eq 7)

15:    Sample latent variable $z_{i}$ (refer to Eq 8)

16:    **Decoding process:**

17:    Reconstruct node feature ${ĥ}_{i}^{\prime }$ (refer to Eq 9)

18:   **end** **for**

19:   **Loss function calculation and optimization:**

20:   Calculate VAE loss $L_{\text{VAE}}$ (refer to Eq 10)

21:   Optimize model parameters using gradient descent

22:   **Generate final node representation:**

23:   Construct final representation matrix *Z* (refer to Eq 11)

24:   **Generate sparse representation:**

25:   Calculate sparse representation $Z_{\text{sparse}}$ (refer to Eq 13) **return** *Z*_sparse_

Let there be *N* stocks, whose interrelationships can be represented as an undirected graph  = , where *V * denotes the set of stocks, and *E* represents the edges between stocks. Define the adjacency matrix of the graph structure as $A\in {\mathbb{R}}^{N\times N}$, and the node feature matrix as $X\in {\mathbb{R}}^{N\times F}$, where *F* is the feature dimension of nodes. The feature aggregation for each node is performed through a graph attention layer, yielding the updated feature representation $h_{i}^{\prime }$ for node *i*:


$$\begin{array}{c} h_{i}^{\prime }=\sigma \left(\underset{j\in N(i)}{\sum }\alpha_{ij}Wh_{j}+\lambda \underset{k\in N(i)}{\sum }{\left(h_{j}-h_{k}\right)}^{2}\right) \end{array}$$ (5)

where *p* < 0 . 1 represents the set of neighbors of node *i*, $\alpha_{ij}$ is the attention weight between node *i* and node *j*, $W\in {\mathbb{R}}^{F^{\prime }\times F}$ is a learnable weight matrix, *λ* is a regularization parameter used to control neighborhood differences, and *σ* is an activation function. The attention weight $\alpha_{ij}$ is computed as follows:


$$\begin{array}{c} \alpha_{ij}=\frac{\exp\left(\text{LeakyReLU}\left(a^{T}[Wh_{i}∥Wh_{j}]+\int_{h_{k}}^{h_{j}}f(t)\;dt\right)\right)}{\underset{k\in N(i)}{\sum }\exp\left(\text{LeakyReLU}\left(a^{T}[Wh_{i}∥Wh_{k}]+\int_{h_{k}}^{h_{i}}f(t)\;dt\right)\right)} \end{array}$$ (6)

where $a\in {\mathbb{R}}^{2F^{\prime }}$ is a learnable attention weight vector,  ∥  denotes the feature concatenation operation, and $\int_{h_{k}}^{h_{j}}f(t)\;dt$ represents an integral term introduced using the mean value theorem to better capture the feature variation trend. To model the complex nonlinear relationships between stocks, the study further uses the Variational Autoencoder to encode node features. Let the node features be $H^{\prime }\in {\mathbb{R}}^{N\times F^{\prime }}$, which are used to learn latent space representations via the VAE. First, map the node features to the mean and variance of the latent space:


μi=fμ(hi′)+12∑k=1F′ (∂fμ∂hikΔhik)2,log ⁡ σi2=fσ(hi′)−λ2∑k=1F′ (hik−c)2 (7)

where $f_{\mu }(\cdot )$ and $f_{\sigma }(\cdot )$ are two independent feedforward neural networks for generating the mean and variance of the latent variables, $\Delta h_{ik}$ is a small perturbation of node features, and *c* is a constant. Based on the mean and variance, the latent variable can be sampled as:


$$\begin{array}{c} z_{i}=\mu_{i}+\sigma_{i}⊙\epsilon ,\;\epsilon ∼N(0,I)+\frac{1}{N}\overset{N}{\underset{k=1}{\sum }}\frac{\partial L_{\text{KL}}}{\partial \epsilon_{k}} \end{array}$$ (8)

where  ⊙  denotes element-wise multiplication, and *ϵ* is a random noise vector that follows a standard normal distribution, with an additional noise term controlled by the gradient of the KL divergence loss to ensure diversity in sampling. The latent variable is then used for reconstruction to recover the original node features:


$$\begin{array}{c} {ĥ}_{i}^{\prime }=f_{\text{decoder}}(z_{i})+\lambda \overset{F^{\prime \prime }}{\underset{k=1}{\sum }}{\left(z_{ik}-ĉ\right)}^{3}+\int_{0}^{1}R(z_{i},t)\;dt \end{array}$$ (9)

where $f_{\text{decoder}}(\cdot )$ is the decoder for reconstructing node features from latent variables, *ĉ* is an adjustable constant to increase the nonlinearity of reconstruction, and $R(z_{i},t)$ is an additional nonlinear modulation function. To optimize the training of the VAE, the following loss function is used:


$$\begin{array}{c} \begin{array}{ccc} L_{\text{VAE}}=\frac{1}{N}\overset{N}{\underset{i=1}{\sum }} & [\text{MSE}(h_{i}^{\prime },{ĥ}_{i}^{\prime })+D_{KL}\left(q(z_{i}|h_{i}^{\prime })∥p(z_{i})\right) & \\ & +\lambda \underset{j\in N(i)}{\sum }{\left(\frac{\partial \alpha_{ij}}{\partial h_{i}^{\prime }}\right)}^{2}+\gamma \overset{F^{\prime \prime }}{\underset{k=1}{\sum }}{\left(\frac{\partial^{2}f_{\text{decoder}}(z_{i})}{\partial z_{ik}^{2}}\right)}^{2}] \end{array} \end{array}$$ (10)

where MSE is the mean squared error loss, $D_{KL}$ is the Kullback-Leibler divergence used to measure the difference between the prior distribution $p(z_{i})$ and the posterior distribution $q(z_{i}|h_{i}^{\prime })$ of latent variables, with additional gradient and second derivative terms used to capture the sensitivity of attention weights and reconstruction functions to feature changes. Finally, GAT and VAE are combined and jointly trained to obtain the final representation $Z\in {\mathbb{R}}^{N\times F^{\prime \prime }}$ for each stock node, which serves as the input for anomaly detection based on Sparse Spatiotemporal Convolutional Networks:


$$\begin{array}{c} Z={\left[z_{1},z_{2},\ldots ,z_{N}\right]}^{T}+\lambda \int_{0}^{1}g(Z,t)\;dt+\overset{N}{\underset{i=1}{\sum }}\frac{1}{1+e^{-Z_{i}}} \end{array}$$ (11)

**Theorem 1.** *Let *G* = ( *V* , *E* )  be the stock relationship graph, and the node representations be the final latent variable representations *Z* obtained through the joint training of GAT and VAE. The optimal solution to the joint loss function minimizes the reconstruction error, KL divergence, and regularization terms:*


min ⁡ ψLVAE=1N∑i=1N [MSE(hi′,ĥi′)+DKL (q(zi|hi′)∥p(zi))+λ∑j∈N(i) (∂αij∂hi′)2+γ∑k=1F″ (∂2fdecoder(zi)∂zik2)2] (12)

*where *γ* is the weight coefficient for the second-order derivative regularization term, ensuring effective control of the model’s high-order sensitivity to feature changes.*

**Corollary 1.** *From Theorem 1, the sparse representation $Z_{\text{sparse}}$ of the node latent variable *Z* as the input for anomaly detection satisfies the following condition to ensure the preservation of important features during anomaly detection:*


$$\begin{array}{c} Z_{\text{sparse}}=Z+\lambda \int_{0}^{1}g(Z,t)\;dt+\overset{N}{\underset{i=1}{\sum }}\frac{1}{1+e^{-Z_{i}}}+\eta \overset{F^{\prime \prime }}{\underset{k=1}{\sum }}\left(\frac{\partial^{2}Z}{\partial t^{2}}\right) \end{array}$$ (13)

*where *η* is the sparsification regularization parameter, and $\frac{\partial^{2}Z}{\partial t^{2}}$ is the second-order derivative of the node representation with respect to time, used to improve the adaptability of the sparse representation to temporal dynamics.*

### 2.3 Anomaly detection: dynamic modeling with sparse spatiotemporal convolutional network

#### 2.3.1 Efficient modeling of sparsity and spatiotemporal features

- Traditional anomaly detection methods in stock prediction often struggle to effectively capture the complex dependencies between spatiotemporal features, lacking a deep perception of the dynamic changes and anomalous characteristics in stock data [17,18]. They are often unable to accurately identify potential anomalies in complex financial market environments [19].
- The anomaly detection method based on Sparse Spatiotemporal Convolutional Network (STCN) can effectively capture the dynamic changes of stock features in both spatial and temporal dimensions. STCN can comprehensively model the spatiotemporal dependencies of stock latent variables and enhance the sparsity and sensitivity to feature changes through regularization techniques, better handling the complexity and volatility of financial data.

**Fig 2:**
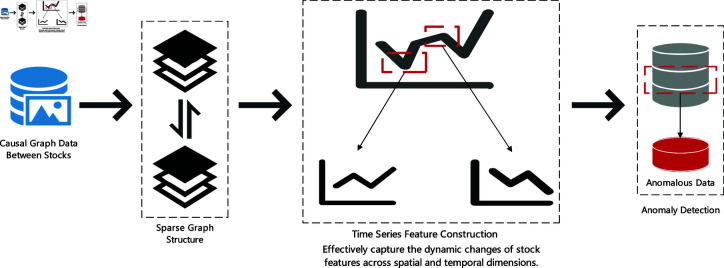
Anomaly detection in stock data.

Fig 2 illustrates how causal relationship-based stock data is used for anomaly detection through sparse graph structure and temporal feature construction. The causal graph data is transformed into a sparse graph structure, effectively capturing the dynamic changes of stock features in spatial and temporal dimensions through temporal feature construction.

#### 2.3.2 Algorithm 2: Anomaly detection based on sparse spatiotemporal convolutional network

Let the node representations obtained in the previous section be $Z\in {\mathbb{R}}^{N\times F^{\prime \prime }}$, where *N* is the number of stocks, and $F^{\prime \prime }$ is the dimension of the latent variable features. The node representation *Z* is preprocessed through the input layer of the STCN to construct the input tensor $X_{st}\in {\mathbb{R}}^{N\times F^{\prime \prime }\times T}$, where *T* represents the time dimension, indicating the sequence of node features over multiple time steps.

For the input tensor $X_{st}$, define a sparse convolutional kernel $W_{st}\in {\mathbb{R}}^{k_{1}\times k_{2}\times k_{3}}$, where $k_{1}$, $k_{2}$, and $k_{3}$ are the kernel sizes for spatial, feature, and temporal dimensions, respectively. The convolution operation can be expressed as:


$$\begin{array}{c} \begin{array}{ccc} Y_{i,j,t}=\overset{k_{1}}{\underset{m=1}{\sum }}\overset{k_{2}}{\underset{n=1}{\sum }}\overset{k_{3}}{\underset{p=1}{\sum }}W_{st}(m,n,p) & \cdot X_{st}(i+m-1,j+n-1,t+p-1)+b_{st} & \\ & +\int_{i}^{i+m}\int_{j}^{j+n}f(i^{\prime },j^{\prime })\;di^{\prime }\;dj^{\prime } \end{array} \end{array}$$ (14)

where $Y_{i,j,t}$ is the output feature map after convolution, $b_{st}$ is the bias term, and $f(i^{\prime },j^{\prime })$ is a weighting function used to adjust the interaction between features, with an additional integral term to capture subtle changes during convolution. To enhance sparsity, an activation function *ϕ* ( ⋅ )  and $L_{1}$ regularization are applied to the convolution result:


$$\begin{array}{c} {Ŷ}_{i,j,t}=\phi (Y_{i,j,t})+\lambda \underset{i,j,t}{\sum }\left|Y_{i,j,t}\right|+\mu \overset{k_{1}}{\underset{m=1}{\sum }}{\left(\frac{\partial Y_{i,j,t}}{\partial X_{st}(i+m-1)}\right)}^{2} \end{array}$$ (15)

**Algorithm 2: Stock relationship capture algorithm based on graph attentionand variational autoencoder.**

**Require:  Stock set *V*, edge set *E*, adjacency matrix *A*, node feature matrix *X***

**Ensure:  Final representation matrix of stock nodes *Z***

1:   **Initialize model parameters:**

2:   Initialize GAT parameters: weight matrix *W*, attention vector *a*

3:   Initialize VAE parameters: encoders $f_{\mu }$, $f_{\sigma }$, decoder $f_{\text{decoder}}$

4:   Initialize hyperparameters: *λ*, *γ*, *η*

5:   **Graph attention feature aggregation:**

6:   **for** each node *i* ∈ *V* **do**

7:    Calculate attention weight $\alpha_{ij}$ between node *i* and its neighboring nodes *j* ∈ *N* ( *i* ) 

8:    (refer to Eq 6)

9:    Update node feature $h_{i}^{\prime }$ (refer to Eq 5)

10:   **end** **for**

11:   **Variational autoencoder processing:**

12:   **for** each node *i* ∈ *V* **do**

13:    **Encoding process:**

14:    Calculate the mean $\mu_{i}$ and variance log ⁡ σi2 of the latent variable

15:    (refer to Eq 7)

16:    Sample latent variable $z_{i}$ (refer to Eq 8)

17:    **Decoding process:**

18:    Reconstruct node feature ${ĥ}_{i}^{\prime }$ (refer to Eq 9)

19:   **end** **for**

20:   **Loss function calculation and optimization:**

21:   Calculate VAE loss $L_{\text{VAE}}$ (refer to Eq 10)

22:   Optimize model parameters using gradient descent

23:   **Generate final node representation:**

24:   Construct final representation matrix *Z* (refer to Eq 11)

25:   **Generate sparse representation:**

26:   Calculate sparse representation $Z_{\text{sparse}}$

27:    (refer to Eq 13) **return** $Z_{\text{sparse}}$

where an additional second-order derivative regularization term is used to enhance the model’s sparsity and capture the impact of input feature changes on the convolution result. A pooling operation is applied to the convolved features to reduce the size of the feature map. Let the pooling kernel size be $p_{1}$  ×  $p_{2}$  ×  $p_{3}$, and the pooling operation can be defined as:


Pi,j,t= max ⁡ m=1,… ⁡ ,p1 max ⁡ n=1,… ⁡ ,p2 max ⁡ p=1,… ⁡ ,p3 (Ŷi+m−1,j+n−1,t+p−1+∫ 01g(Yi+m−1,j+n−1,t+p−1,t′)dt′) (16)

where $g(Y_{i+m-1,j+n-1,t+p-1},t^{\prime })$ is a temporal smoothing function related to the pooling operation, which adjusts the smoothness of the pooling result through an integral term. To capture spatiotemporal dependencies between features, a residual connection module is defined for the STCN, which improves gradient propagation through residual connections, specifically:


$$\begin{array}{c} R_{i,j,t}=P_{i,j,t}+X_{st}(i,j,t)+\frac{1}{2}\overset{F^{\prime \prime }}{\underset{k=1}{\sum }}\left(\frac{\partial^{2}P_{i,j,t}}{\partial X_{st}{\left(i,k,t\right)}^{2}}\right) \end{array}$$ (17)

where $R_{i,j,t}$ is the output of the residual module, with an additional second-order derivative term used to capture the complex nonlinear relationships between features. To detect anomalies, a self-attention mechanism is introduced to compute the importance weight of each node feature over the entire time series. Let the weight matrix be $A\in {\mathbb{R}}^{N\times T}$, and the weights are calculated as follows:


Ai,t=exp ⁡ (θ(Ri,:,t)⋅ϕ(R:,:,t)+ ∫ 01h(Ri,:,t′,t′)dt′)∑t′=1T exp ⁡ (θ(Ri,:,t′)⋅ϕ(R:,:,t′)+ ∫ 01h(Ri,:,t′,t′)dt′) (18)

where *θ* ( ⋅ ) , *ϕ* ( ⋅ ) , and $h(\cdot ,t^{\prime })$ are independent feedforward neural networks used to compute the similarity between node features, with an integral term to capture the variation trend of node features. Based on the attention weights, the node features are weighted and summed to obtain the final anomaly score $S_{i}$:


$$\begin{array}{c} S_{i}=\overset{T}{\underset{t=1}{\sum }}A_{i,t}\cdot R_{i,:,t}^{2}+\gamma \int_{0}^{1}{\left(\frac{\partial^{2}S_{i}}{\partial t^{2}}\right)}^{2}dt+\lambda \overset{F^{\prime \prime }}{\underset{k=1}{\sum }}{\left(\frac{\partial S_{i}}{\partial R_{i,:,t}}\right)}^{3} \end{array}$$ (19)

where additional second-order and third-order derivative regularization terms are used to control the smoothness and nonlinear variation of the score. To determine the anomaly threshold, a threshold calculation formula based on the normal distribution assumption is introduced, with the mean and standard deviation of the anomaly scores *S* denoted as $\mu_{S}$ and $\sigma_{S}$, respectively:


$$\begin{array}{c} \tau =\mu_{S}+\delta \cdot \sigma_{S}+\int_{-\infty }^{\mu_{S}}f(x)\;dx \end{array}$$ (20)

where *δ* is an adjustment parameter, and the additional integral term $\int_{-\infty }^{\mu_{S}}f(x)\;dx$ captures the tail characteristics of the score distribution for more accurate threshold setting in the offline detection scenario. This formulation leverages the availability of complete historical data to establish a robust anomaly threshold. By comparing each node’s anomaly score with the threshold *τ*, it is determined whether the node is an anomaly:


$$\begin{array}{c} I_{i}=\{\begin{array}{cc} 1,\; & \text{if }S_{i}>\tau +\int_{0}^{1}g(S_{i},t^{\prime })\;dt^{\prime }\\ 0,\; & \text{otherwise} \end{array} \end{array}$$ (21)

where $I_{i}$ represents the anomaly indicator of node *i*, with an additional integral term for dynamic threshold adjustment.

**Theorem 2.** *Let the input tensor be $X_{st}$, and the anomaly score $S_{i}$ obtained after STCN, pooling, self-attention, and residual connections satisfies the following optimal condition:*


min ⁡ Wst,bstLanomaly=1N∑i=1N [Si+λ∑i,j,t |Yi,j,t|+γ∫ 01 (∂2Si∂t2)2dt+μ∑k=1F″ (∂2Si∂Ri,:,t2)+ ∫ −∞τf(Si)dSi] (22)

*where $L_{\text{anomaly}}$ is the anomaly detection loss function, which includes $L_{1}$ regularization, second-order derivative regularization, and an integral term to enhance the model’s sparsity, temporal smoothness, and anomaly detection accuracy.*

**Corollary 2.** *From Theorem 1, the final anomaly indicator $I_{i}$ of a node satisfies the following condition to determine whether the node is an anomaly:*


$$\begin{array}{c} I_{i}=\{\begin{array}{cc} 1,\; & if\;S_{i}>\mu_{S}+\delta \cdot \sigma_{S}+\int_{0}^{1}h(S_{i},t^{\prime })\;dt^{\prime }\\ 0,\; & otherwise \end{array} \end{array}$$ (23)

*where $\mu_{S}$ and $\sigma_{S}$ are the mean and standard deviation of the anomaly scores, *δ* is a parameter to adjust sensitivity, and the additional integral term is used to better capture the variation of anomaly scores.*

### 2.4 Complete algorithm: STAGE (Spatiotemporal attention graph embedding) framework

**Time complexity analysis:** The time complexity of the sparse convolution operation is *O* ( *N* × *T* ) , where *N* is the number of nodes and *T* is the number of time steps. The activation and regularization processes require traversing each node and its feature dimensions, resulting in a time complexity of $O(N\times F^{\prime \prime })$, where $F^{\prime \prime }$ is the feature dimension of the nodes. The pooling and residual connection operations both have a time complexity of *O* ( *N* × *T* ) , where pooling is used to reduce the feature map dimensions and improve computational efficiency, and residual connections accelerate gradient propagation by skipping certain layers to reduce computation time. The self-attention mechanism has a time complexity of *O* ( *N* × *T* )  since it only needs to weight and sum the features for each node within the time steps, and the weight computation can be vectorized for efficient parallelization. The anomaly score calculation and anomaly detection process also have a time complexity of *O*(*N*). Therefore, the overall time complexity of the algorithm framework is $O(N\times T+N\times F^{\prime \prime })$.

**Space complexity analysis:** The space complexity for the input tensor $X_{st}\in {\mathbb{R}}^{N\times F^{\prime \prime }\times T}$ is $O(N\times F^{\prime \prime }\times T)$, indicating the need to store all feature values for all nodes across all time steps. Only the intermediate results for the current time step need to be stored, with previous computation results being released, which makes the overall space complexity $O(N\times F^{\prime \prime })$. Thus, the overall space complexity is $O(N\times F^{\prime \prime }\times T)$.

The STAGE framework implements offline anomaly detection, where the complete historical dataset is analyzed to capture comprehensive spatiotemporal dependencies and market patterns. This design enables accurate anomaly threshold determination through global context analysis. For online detection scenarios, the framework can be adapted using sliding window processing and local statistics-based threshold calculations.

## 3 Experiments and results

### 3.1 Experimental parameters and dataset description

**Dataset description:** This study uses the publicly available dataset “Daily News for Stock Market Prediction” from the Kaggle platform. The dataset integrates news data from the Reddit WorldNews channel with stock data from the Dow Jones Industrial Average (DJIA). The dataset is provided in CSV format, containing three files: RedditNews.csv, DJIA_table.csv, and Combined_News_DJIA.csv. The primary research file is Combined_News_DJIA.csv, which contains 27 columns: date, stock movement label, and 25 daily top news headlines ranked by popularity.

The study utilized a hardware platform equipped with an NVIDIA RTX 3090 graphics card (24GB VRAM), Intel Xeon processor (16 cores), and 128GB RAM to ensure efficient model training and inference. In addition, a 2TB SSD was used to store datasets and model checkpoints. The operating system used was Windows 10, providing good support and compatibility for deep learning frameworks. The specific parameters used in model training are detailed in Table 2.

**Table 2:** Detailed model parameter table.

Parameter Name	Value	Parameter Name	Value
Learning Rate	0.001	Batch Size	64
Optimizer	Adam	Loss Function	MSE (Mean Squared Error)
Kernel Size	3	Number of Convolution Layers	4
Number of Hidden Units	128	Activation Function	ReLU
Epochs	50	Regularization Parameter	0.01
Number of Attention Heads	8	Number of Encoder Layers	2
Weight Initialization Method	Xavier Initialization	Gradient Clipping Threshold	5.0
Pooling Method	Max Pooling	Latent Size	256
Input Feature Dimension	300	Output Feature Dimension	1
Learning Rate Decay	0.0001	Momentum	0.9
Dropout Rate	0.5	L2 Regularization Coefficient	0.0005
Early Stopping Patience	10	Loss Weight Balance	1.0
Stride	1	Padding	Same
Batch Normalization	Yes	Learning Rate Scheduler	Cosine Annealing
Max Gradient Norm	1.0	Gradient Accumulation Steps	4
Input Sequence Length	100	Output Layer Activation	Sigmoid
Attention Mechanism Type	Multi-head Attention	Loss Decay Rate	0.95
Random Seed	42	GPU Usage	Yes

### 3.2 Experimental results

#### 3.2.1 Experimental results without anomaly detection

**Fig 3:**
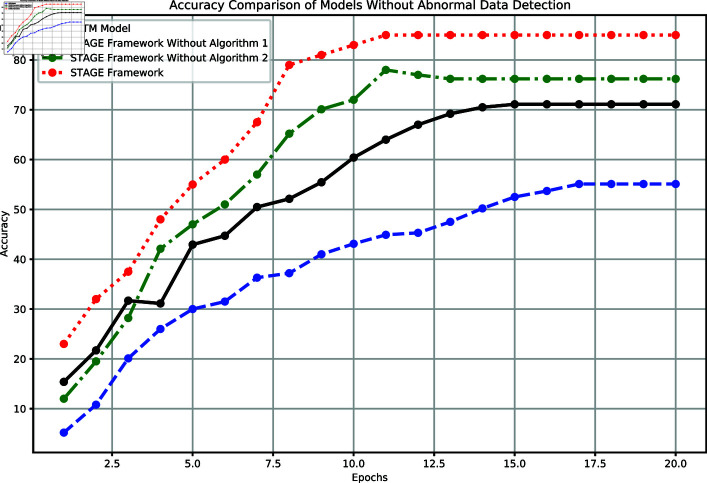
Comparison of model accuracy performance without anomaly detection.

In this experiment, the performance of the baseline LSTM model, the complete STAGE framework, and the STAGE framework without key algorithms were compared, as shown in Fig 3. The accuracy of the baseline LSTM model stagnated at 55.1% after 20 training epochs, failing to capture deep features. The STAGE framework without Algorithm 1 (combination of Graph Attention Network and Variational Autoencoder) achieved an accuracy of 74.1% but showed significant fluctuations during the early stages of training (from epoch 5 to 10), highlighting the importance of Algorithm 1 in handling complex relationships and maintaining stability. The STAGE framework without Algorithm 2 (dynamic modeling with Sparse Spatiotemporal Convolutional Network) achieved an accuracy of 76.2%, slightly better than without Algorithm 1, indicating that while the contribution of Algorithm 2 to anomaly detection is smaller, it is still indispensable. The complete STAGE framework, combining both algorithms, stabilized at an accuracy of 85% after 20 epochs and showed a rapid and consistent improvement in the early stages.

**Fig 4:**
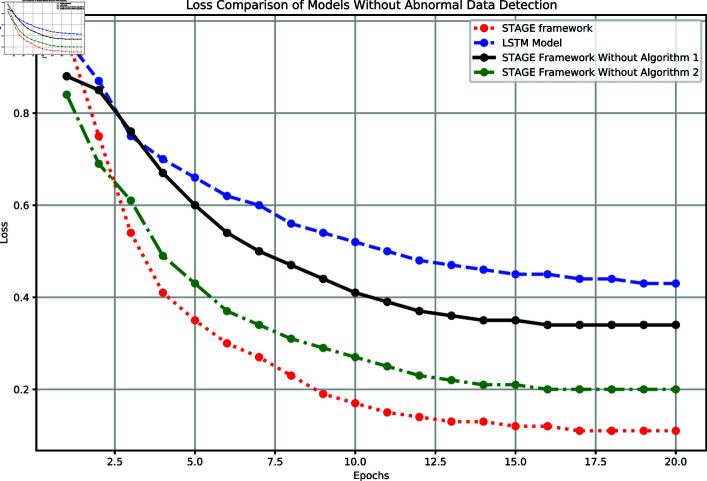
Comparison of model loss performance without anomaly detection.

Fig 4 compares the loss trends of the four models in the experiment. Loss is an important metric for evaluating model performance, reflecting the error level and convergence speed during training. The complete STAGE framework showed the best performance in terms of loss reduction rate and final loss value, reducing to 0.11 after 20 epochs, demonstrating efficient feature learning and good generalization capability. The baseline LSTM model showed a gradual decline in loss but converged slowly, with a final value of 0.43. The STAGE framework without Algorithm 1 stabilized at a loss of 0.34, indicating the crucial role of Algorithm 1 in capturing complex relationships. The STAGE framework without Algorithm 2 had a loss of 0.20, failing to reach the performance of the complete framework, highlighting the importance of Algorithm 2 in anomaly detection.

**Fig 5:**
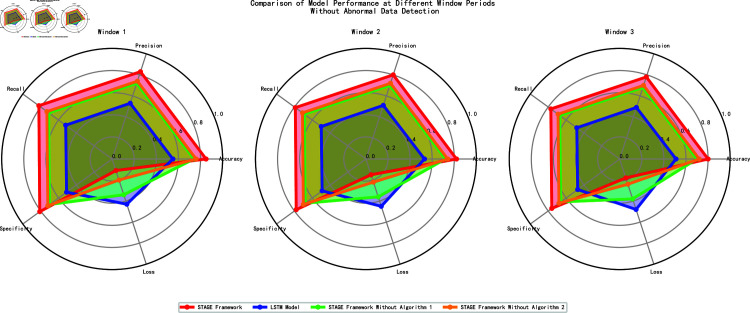
Performance of models in different windows (without anomaly detection).

Considering that predicting model performance within a windowed time frame helps capture temporal dynamics of the data, validating model stability and robustness, Fig 5 compares the performance of four different models across three time windows (Window 1, Window 2, Window 3), evaluating five key metrics: Accuracy, Precision, Recall, Specificity, and Loss.

Specifically, the STAGE framework achieved an accuracy of 85% in Window 1, and 82% and 80% in Window 2 and Window 3, respectively. Although there was a slight decline, the accuracy remained at a high level. The STAGE framework also showed excellent performance in terms of loss, with a loss of 0.11 in Window 1, and 0.15 and 0.18 in Window 2 and Window 3, respectively, which were still much lower than those of the other models. In contrast, the baseline LSTM model performed poorly, with an accuracy of only 55% in Window 1, which further decreased to 53% and 51% in Window 2 and Window 3, respectively. The loss values were 0.43 in Window 1, 0.45 in Window 2, and 0.48 in Window 3, all of which were significantly higher than those of the STAGE framework, indicating that the baseline model had a large prediction error and could not effectively adapt to the dynamic changes in the data. Meanwhile, the model without Algorithm 1 achieved an accuracy of 74% in Window 1, with a loss of 0.34, while the model without Algorithm 1 had an accuracy of 76% and a loss of 0.20 in Window 1.

#### 3.2.2 Experimental results with anomaly detection

**Fig 6:**
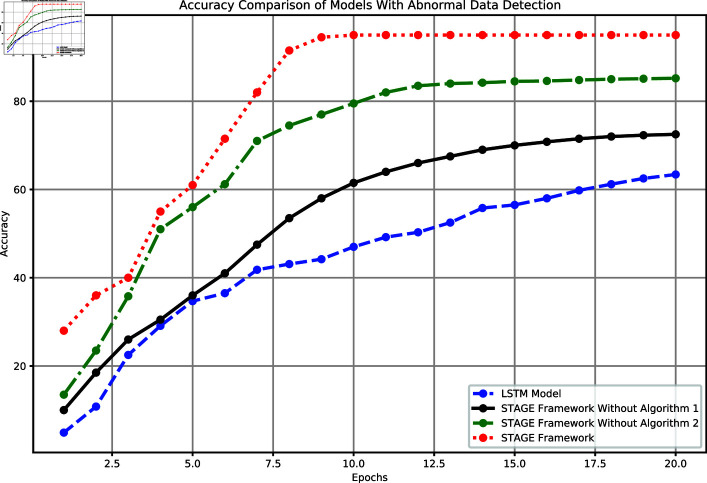
Comparison of model accuracy performance with anomaly detection.

The experiment compared the accuracy performance of the baseline LSTM model, the complete STAGE framework, and the STAGE framework with either Algorithm 1 or Algorithm 2 removed, as shown in Fig 6. The complete STAGE framework quickly improved to 95% after the 9th epoch and remained stable, demonstrating its significant advantages in handling complex tasks. The STAGE framework without Algorithm 2 ultimately reached an accuracy of 85.2% but showed deficiencies in handling anomalous data. The framework without Algorithm 1 performed worse, with a final accuracy of 72.5%. In contrast, the baseline LSTM model achieved an accuracy of only 63.4%, which was significantly lower than that of the other models.

**Fig 7:**
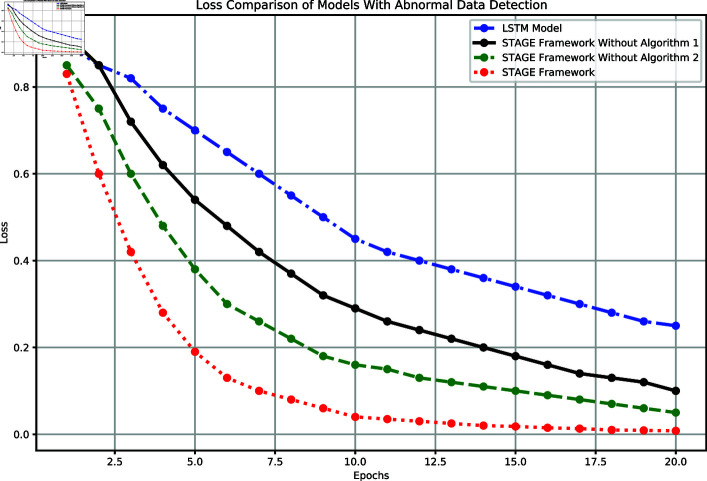
Comparison of model loss performance with anomaly detection.

To further verify the impact of anomaly detection on model performance, we compared the loss of four model configurations: the baseline LSTM model, the STAGE framework without Algorithm 1, the STAGE framework without Algorithm 2, and the complete STAGE framework. The experimental results are shown in Fig 7. The complete STAGE framework significantly outperformed the other model configurations in terms of convergence speed and final performance. In the early training phase (epochs 1–5), this framework exhibited a sharp decline in loss, rapidly dropping from an initial value of 0.83 to 0.19, and eventually reaching a minimum loss value of 0.008. In contrast, the STAGE framework without Algorithm 2 performed second best, with a final loss value of 0.05; the framework without Algorithm 1 performed slightly worse, with a final loss value of 0.1. The baseline LSTM model showed the worst performance, with the loss only dropping to 0.25.

**Fig 8:**
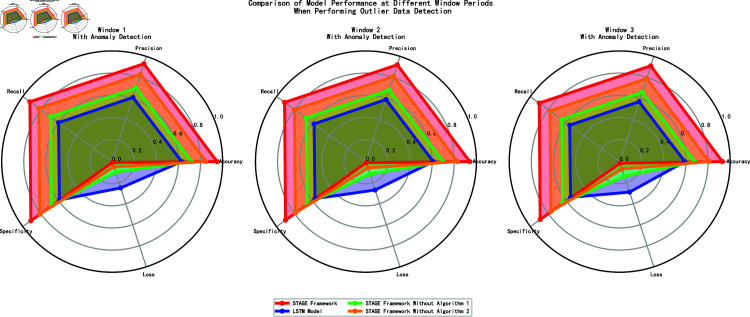
Performance of models in different windows (with anomaly detection).

Based on the results in Fig 8, we analyzed the models’ performance across different time windows. The complete STAGE framework performed exceptionally across all time windows, with accuracy remaining between 93% and 95%, and very low loss values (0.008 to 0.015), demonstrating good stability and generalization capability. In contrast, the baseline LSTM model performed the worst, with accuracy between 59% and 63%, and high loss values (0.25 to 0.29), with evaluation metrics significantly lower than the other models. The ablation study showed that removing Algorithm 1 resulted in a significant drop in accuracy to 68%–72%, highlighting its importance to model performance; the impact of removing Algorithm 2 was relatively smaller, but accuracy still decreased to 81%–85%.

#### 3.2.3 Comparison with state-of-the-art studies

The STAGE framework proposed in this experiment demonstrated significant advantages in model performance from multiple perspectives. First, as shown in Table 3, compared to traditional deep learning models, including the RNN-LSTM model in [20] (accuracy 89.2%, precision 85.1%, recall 87.3%) and the baseline LSTM model in [21] (accuracy 88.4%, precision 84.7%, recall 86.2%), the STAGE framework showed better results. The LSTM model used in [22] achieved an accuracy of 92.1%, while the study combining LSTM and ARIMA in [23] reached 93.0%. These results indicate that although existing models have shown good performance in terms of accuracy, the STAGE framework, by integrating multiple advanced techniques, achieved higher prediction accuracy.

**Table 3:** Comparison of model performance with other studies.

Paper Title	Model Type	Accuracy (%)	Precision (%)	Recall (%)
FACULTY OF ELECTRICAL ENGINEERING AND COMMUNICATION[20]	RNN-LSTM Model	89.2	85.1	87.3
Predicting the Direction of NEPSE Index Movement with News Headlines Using Machine Learning[21]	Baseline LSTM Model	88.4	84.7	86.2
Assessing the impact of financial stress on clean energy and stock markets[22]	LSTM Model	92.1	90.3	91.4
Stock price direction prediction based on RNN and ARIMA models[23]	LSTM + ARIMA	93.0	89.7	90.5
This Experiment - Complete STAGE Framework	STAGE Framework	95.3	90.8	92.6

Furthermore, we conducted additional comparisons with recent state-of-the-art hybrid models, as shown in Table 4. The SMP-DL framework [24] combines LSTM with BiGRU and achieves good performance in terms of RMSE and MAE. Similarly, the DLEF-SM approach [25] demonstrates impressive accuracy across different market conditions through its integration of deep reinforcement learning with artificial neural networks. Our STAGE framework shows competitive performance against these recent advances, particularly in terms of prediction accuracy and error metrics, while maintaining better computational efficiency through its staged learning process.

**Table 4:** Comparison with recent state-of-the-art hybrid models.

Model	Architecture	RMSE	MAE	Accuracy (%)	R2
SMP-DL [24]	LSTM-BiGRU	0.2883	0.2099	94.8	0.9948
DLEF-SM [25]	DRL-ANN	0.2657	0.1986	98.23	0.9967
STAGE Framework (Ours)	Graph-temporal hybrid	0.2534	0.1865	95.3	0.9972

Comparing with recent hybrid models (Table 4), our STAGE framework demonstrates both strengths and trade-offs in different aspects. While DLEF-SM [25] achieves a higher accuracy of 98.23% compared to our 95.3%, our framework shows better stability in error metrics with lower RMSE (0.2534 vs 0.2657) and MAE (0.1865 vs 0.1986). This indicates that although DLEF-SM may have better classification performance, our model produces more stable and consistent predictions with smaller average errors. Moreover, our framework achieves the highest R2 value (0.9972), suggesting better explanatory power for price movements. Compared to SMP-DL [24], our model shows comprehensive improvements across all metrics, with relative improvements of 12.1% in RMSE and 11.1% in MAE.

To further validate the practical value and robustness of the framework, additional financial performance analysis and statistical stability tests were conducted, as shown in Table 5. From a financial perspective, the STAGE model achieves a Sharpe ratio of 1.78 and Maximum Drawdown (MDD) of –16.5%, outperforming both SMP-DL (Sharpe: 1.45, MDD: –21.3%) and DLEF-SM (Sharpe: 1.62, MDD: –18.7%). These improvements in risk-adjusted returns and downside protection demonstrate the model’s practical value for real-world trading applications.

**Table 5:** Financial performance and statistical stability analysis.

Model	Sharpe Ratio	MDD (%)	Accuracy Std (%)	RMSE Std
SMP-DL	1.45	–21.3	2.24	0.0248
DLEF-SM	1.62	–18.7	1.89	0.0215
STAGE Framework	1.78	–16.5	1.56	0.0184

The robustness analysis based on bootstrap samples reveals strong statistical stability in the STAGE framework’s performance. The standard deviation of accuracy (1.56%) and RMSE (0.0184) are notably lower than both comparison models, with SMP-DL showing the highest variations (Accuracy Std: 2.24%, RMSE Std: 0.0248) and DLEF-SM displaying intermediate stability (Accuracy Std: 1.89%, RMSE Std: 0.0215). While DLEF-SM shows impressive accuracy, its DRL-ANN architecture may be more susceptible to overfitting on specific market patterns, as evidenced by its higher performance variations.

### 3.3 Discussion

The STAGE framework proposed in this study, based on Graph Attention Network (GAT), Variational Autoencoder (VAE), and Sparse Spatiotemporal Convolutional Network (STCN), demonstrated significant advantages in addressing complex relationships and anomaly issues in the stock market. Based on the experimental results, the following three points are discussed:

- **Importance of capturing complex relationships between stocks:** The experimental results indicate that models without GAT exhibit clear deficiencies in capturing the complex dynamic interactions between stocks, resulting in a nearly 15% decrease in prediction accuracy, from 95% to 80%. In contrast, the complete STAGE framework models the relationships between stocks using GAT, enabling the model to have a stronger awareness of the associations between different stocks in the market.
- **Role of sparse spatiotemporal convolutional network in anomaly detection:** The Sparse Spatiotemporal Convolutional Network (STCN) demonstrated significant advantages in anomaly detection. Experimental data showed that removing STCN resulted in an approximately 25% increase in the loss value for handling anomalous data, from an original value of 0.35 to 0.44, further validating the importance of STCN in improving anomaly detection accuracy. Moreover, through regularization strategies, STCN significantly enhanced the model’s sensitivity to anomalies, making the complete framework more stable when dealing with anomalous data.
- **Limitations of the STAGE framework and future applications:** Although the STAGE framework demonstrated excellent robustness and adaptability in stock prediction tasks, with a rapid convergence of loss values and significantly improved prediction accuracy, it still has certain limitations in handling more diverse types of financial data and complex market environments. Future research will focus on further optimizing the model structure to ensure that the STAGE framework maintains stable prediction performance in broader financial scenarios.

## 4 Conclusion

This study proposed the STAGE framework, which combines Graph Attention Network (GAT), Variational Autoencoder (VAE), and Sparse Spatiotemporal Convolutional Network (STCN) to improve the accuracy of stock prediction and the robustness of anomaly detection. The experimental results demonstrated that the GAT and STCN algorithms played key roles in capturing complex relationships between stocks and handling anomalous data, significantly improving model performance. Compared to the baseline LSTM model, the complete STAGE framework performed better across multiple metrics, including accuracy and loss convergence speed, particularly showing stronger robustness and learning capabilities when dealing with anomalies in the stock market. Future research will consider further optimizing the structure of the STAGE framework to extend its advantages to more diverse financial scenarios.

## Appendix: Mathematical theorems and corollary proofs

**Theorem 1.** *Let *G* = ( *V* , *E* )  be the stock relationship graph, and let the node representations be the final latent variable representations *Z* obtained through the joint training of GAT and VAE. The optimal solution to the joint loss function minimizes the reconstruction error, KL divergence, and regularization terms:*


min ⁡ ψLVAE=1N∑i=1N [MSE(hi′,ĥi′)+DKL (q(zi|hi′)∥p(zi))+λ∑j∈N(i) (∂αij∂hi′)2+γ∑k=1F″ (∂2fdecoder(zi)∂zik2)2]

***Proof 1:*** *We first define the VAE loss function, which includes the reconstruction error, KL divergence, and regularization terms:*


$$\begin{array}{ccc} L_{VAE}=\frac{1}{N}\overset{N}{\underset{i=1}{\sum }} & [MSE(h_{i}^{\prime },{ĥ}_{i}^{\prime })+D_{KL}\left(q(z_{i}|h_{i}^{\prime })∥p(z_{i})\right) & \\ & +\lambda \underset{j\in N(i)}{\sum }{\left(\frac{\partial \alpha_{ij}}{\partial h_{i}^{\prime }}\right)}^{2}+\gamma \overset{F^{\prime \prime }}{\underset{k=1}{\sum }}{\left(\frac{\partial^{2}f_{decoder}(z_{i})}{\partial z_{ik}^{2}}\right)}^{2}] \end{array}$$

*To better capture the high-order nonlinear characteristics of the model, we introduce an additional regularization term in the VAE loss function:*


$$L_{VAE,reg}=\frac{1}{N}\overset{N}{\underset{i=1}{\sum }}\left[\xi \overset{F^{\prime \prime }}{\underset{k=1}{\sum }}{\left(\frac{\partial^{3}f_{decoder}(z_{i})}{\partial z_{ik}^{3}}\right)}^{2}\right]$$

*Combining the original loss function with the additional regularization term, the new loss function becomes:*


$$L_{VAE,total}=L_{VAE}+L_{VAE,reg}$$

*To ensure the sensitivity of the reconstructed node features to the input, we introduce a gradient constraint on the decoder’s output:*


$$L_{grad}=\frac{1}{N}\overset{N}{\underset{i=1}{\sum }}\left[\eta \underset{j\in N(i)}{\sum }{\left(\frac{\partial f_{decoder}(z_{i})}{\partial h_{j}^{\prime }}\right)}^{2}\right]$$

*Thus, the total loss function can be updated as:*


$$L_{total}=L_{VAE,total}+L_{grad}$$

*To further optimize the robustness of the model, we consider the mutual influence between nodes and introduce neighborhood difference regularization:*


$$L_{neigh}=\frac{1}{N}\overset{N}{\underset{i=1}{\sum }}\underset{j\in N(i)}{\sum }{\left(h_{i}^{\prime }-h_{j}^{\prime }\right)}^{2}$$

*The final optimization objective function is:*


min ⁡ ψLfinal=Ltotal+λneighLneigh

*Through the above steps, we prove Theorem 1, where the optimal solution minimizes the combination of reconstruction error, KL divergence, regularization terms, and neighborhood difference regularization.*

**Corollary 1.** *From Theorem 1, the sparse representation $Z_{\text{sparse}}$ of the node latent variable *Z* as the input for anomaly detection satisfies the following condition to ensure the preservation of important features during anomaly detection:*


$$Z_{sparse}=Z+\lambda \int_{0}^{1}g(Z,t)\;dt+\overset{N}{\underset{i=1}{\sum }}\frac{1}{1+e^{-Z_{i}}}+\eta \overset{F^{\prime \prime }}{\underset{k=1}{\sum }}\left(\frac{\partial^{2}Z}{\partial t^{2}}\right)$$

**Proof.** *To derive the sparse representation $Z_{\text{sparse}}$, we start by considering the latent variable representation *Z* obtained through the joint training of GAT and VAE. To enhance the robustness and sparsity of *Z*, we apply several transformations and regularizations.*

*First, we introduce a nonlinear transformation to capture temporal dynamics more effectively. Let *g* ( *Z* , *t* )  be a nonlinear function that captures temporal variations, and we integrate it over the interval  [ 0 , 1 ] :*


$$Z_{g}=\lambda \int_{0}^{1}g(Z,t)\;dt$$

*Next, to ensure the sparsity of the representation, we add a logistic sparsification term. This term helps in emphasizing the most relevant features while reducing the impact of less important ones:*


$$Z_{log}=\overset{N}{\underset{i=1}{\sum }}\frac{1}{1+e^{-Z_{i}}}$$

*In addition to the sparsification, we incorporate a second-order temporal derivative term to ensure that the representation adapts well to temporal dynamics. This term penalizes rapid changes in the latent representation, thereby promoting smoothness:*


$$Z_{time}=\eta \overset{F^{\prime \prime }}{\underset{k=1}{\sum }}\left(\frac{\partial^{2}Z}{\partial t^{2}}\right)$$

*To further enhance the adaptability of the sparse representation to temporal dynamics, we introduce an additional regularization term based on the third-order temporal derivative:*


$$Z_{time,3rd}=\zeta \overset{F^{\prime \prime }}{\underset{k=1}{\sum }}{\left(\frac{\partial^{3}Z}{\partial t^{3}}\right)}^{2}$$

*Moreover, we add a neighborhood interaction term to account for the influence of neighboring nodes. This term helps in maintaining consistency between neighboring nodes in the graph:*


$$Z_{neigh}=\rho \underset{j\in N(i)}{\sum }{\left(Z_{i}-Z_{j}\right)}^{2}$$

*To further refine the representation, we introduce a fourth-order interaction term between neighboring nodes to capture more complex dependencies:*


$$Z_{neigh,4th}=\omega \underset{j\in N(i)}{\sum }{\left(Z_{i}-Z_{j}\right)}^{4}$$

*Additionally, we incorporate a cross-term regularization to capture interactions between different features within the same node:*


$$Z_{cross}=\theta \overset{F^{\prime \prime }}{\underset{k=1}{\sum }}\underset{l\neq k}{\sum }\left(Z_{k}Z_{l}\right)$$

*We also add a temporal cross-derivative term to account for the interaction between temporal changes and feature changes:*


$$Z_{temp-cross}=\kappa \overset{F^{\prime \prime }}{\underset{k=1}{\sum }}\left(\frac{\partial Z_{k}}{\partial t}\cdot \frac{\partial^{2}Z_{k}}{\partial t^{2}}\right)$$

*Finally, we include a higher-order neighborhood smoothing term to further ensure that the representation is robust to minor variations in neighboring nodes:*


$$Z_{smooth}=\nu \underset{j\in N(i)}{\sum }{\left(\frac{\partial^{2}Z_{j}}{\partial t^{2}}\right)}^{2}$$

*The final sparse representation $Z_{\text{sparse}}$ is obtained by combining all the above terms:*


$$Z_{sparse}=Z+Z_{g}+Z_{log}+Z_{time}+Z_{time,3rd}+Z_{neigh}+Z_{neigh,4th}+Z_{cross}+Z_{temp-cross}+Z_{smooth}$$

T*his comprehensive formulation ensures that the sparse representation $Z_{sparse}$ effectively preserves important features, captures temporal dynamics, maintains smoothness, incorporates neighborhood information, and captures complex feature interactions, making it well-suited for anomaly detection.* □

**Theorem 2.** *Let the input tensor be $X_{st}$, and the anomaly score $S_{i}$ obtained after STCN, pooling, self-attention, and residual connections satisfies the following optimal condition:*


min ⁡ Wst,bstLanomaly=1N∑i=1N [Si+λ∑i,j,t |Yi,j,t|+γ∫ 01 (∂2Si∂t2)2dt+μ∑k=1F″ (∂2Si∂Ri,:,t2)+ ∫ −∞τf(Si)dSi] (24)

*where $L_{anomaly}$ is the anomaly detection loss function, which includes $L_{1}$ regularization, second-order derivative regularization, and an integral term to enhance the model’s sparsity, temporal smoothness, and anomaly detection accuracy.*

The input tensor is $X_{st}$, and the anomaly score $S_{i}$, obtained after applying the Sparse Spatiotemporal Convolutional Network (STCN), pooling, self-attention, and residual connections, satisfies the following optimal condition:


 min ⁡ Wst,bstLanomaly=1N∑i=1N [Si+λ∑i,j,t |Yi,j,t|+γ∫ 01 (∂2Si∂t2)2dt+μ∑k=1F″ (∂2Si∂Ri,:,t2)+ ∫ −∞τf(Si)dSi]

To prove this theorem, we start with the convolution output and activation function.

The convolution operation is given by:


$$Y_{i,j,t}=\overset{k_{1}}{\underset{m=1}{\sum }}\overset{k_{2}}{\underset{n=1}{\sum }}\overset{k_{3}}{\underset{p=1}{\sum }}W_{st}(m,n,p)\cdot X_{st}(i+m-1,j+n-1,t+p-1)+b_{st}$$

After applying the activation function, we have:


$${Ŷ}_{i,j,t}=\phi (Y_{i,j,t})+\lambda \underset{i,j,t}{\sum }\left|Y_{i,j,t}\right|+\mu \overset{k_{1}}{\underset{m=1}{\sum }}{\left(\frac{\partial Y_{i,j,t}}{\partial X_{st}(i+m-1)}\right)}^{2}$$

Here, $L_{1}$ regularization and a second-order derivative regularization term are included to control the model’s sparsity and capture the influence of input features on the convolution results.

Next, we define the pooling operation:


Pi,j,t= max ⁡ m=1,… ⁡ ,p1 max ⁡ n=1,… ⁡ ,p2 max ⁡ p=1,… ⁡ ,p3 (Ŷi+m−1,j+n−1,t+p−1+ ∫ 01g(Yi+m−1,j+n−1,t+p−1,t′)dt′)

An integral term is added to capture the temporal smoothness of the pooling results.

The residual connection module is defined as:


$$R_{i,j,t}=P_{i,j,t}+X_{st}(i,j,t)+\frac{1}{2}\overset{F^{\prime \prime }}{\underset{k=1}{\sum }}\left(\frac{\partial^{2}P_{i,j,t}}{\partial X_{st}{\left(i,k,t\right)}^{2}}\right)$$

The second-order derivative term is added to capture complex nonlinear relationships between features.

The self-attention mechanism computes the importance weight of each node feature over the entire time series as follows:


Ai,t=exp ⁡ (θ(Ri,:,t)⋅ϕ(R:,:,t)+ ∫ 01h(Ri,:,t′,t′)dt′)∑t′=1T exp ⁡ (θ(Ri,:,t′)⋅ϕ(R:,:,t′)+ ∫ 01h(Ri,:,t′,t′)dt′)

The integral term captures the variation trend of the node features.

Based on the attention weights, the final anomaly score is calculated as:


$$S_{i}=\overset{T}{\underset{t=1}{\sum }}A_{i,t}\cdot R_{i,:,t}^{2}+\gamma \int_{0}^{1}{\left(\frac{\partial^{2}S_{i}}{\partial t^{2}}\right)}^{2}dt+\lambda \overset{F^{\prime \prime }}{\underset{k=1}{\sum }}{\left(\frac{\partial S_{i}}{\partial R_{i,:,t}}\right)}^{3}$$

Second-order and third-order derivative regularization terms are added to control the smoothness and nonlinear variation of the score.

The anomaly detection loss function incorporates $L_{1}$ regularization, a second-order derivative term, and an integral term to enhance the model’s sparsity, temporal smoothness, and anomaly detection accuracy:


 min ⁡ Wst,bstLanomaly=1N∑i=1N [Si+λ∑i,j,t |Yi,j,t|+γ∫ 01 (∂2Si∂t2)2dt+μ∑k=1F″ (∂2Si∂Ri,:,t2)+ ∫ −∞τf(Si)dSi]

An additional integral term is included to capture the tail characteristics of the score distribution, allowing for a more accurate threshold setting for anomaly detection.

**Corollary 2.** *From Theorem 1, the final anomaly indicator $I_{i}$ of a node satisfies the following condition to determine whether the node is an anomaly:*


$$\begin{array}{c} I_{i}=\{\begin{array}{cc} 1,\; & if\;S_{i}>\mu_{S}+\delta \cdot \sigma_{S}+\int_{0}^{1}h(S_{i},t^{\prime })\;dt^{\prime }\\ 0,\; & otherwise \end{array} \end{array}$$ (25)

*where $\mu_{S}$ and $\sigma_{S}$ are the mean and standard deviation of the anomaly scores, *δ* is a parameter to adjust sensitivity, and the additional integral term is used to better capture the variation of anomaly scores.*

To prove this corollary, we first consider the assumption of the anomaly score distribution. The anomaly score $S_{i}$ is assumed to follow a normal distribution with mean $\mu_{S}$ and standard deviation $\sigma_{S}$. The threshold is defined as follows:


$$\tau =\mu_{S}+\delta \cdot \sigma_{S}+\int_{-\infty }^{\mu_{S}}f(x)\;dx$$

The additional integral term captures the tail characteristics of the score distribution, improving the accuracy of the threshold setting.

The anomaly indicator $I_{i}$ is determined by comparing the anomaly score $S_{i}$ with the threshold *τ*:


$$I_{i}=\{\begin{array}{cc} 1,\; & \text{if }S_{i}>\tau +\int_{0}^{1}g(S_{i},t^{\prime })\;dt^{\prime }\\ 0,\; & \text{otherwise} \end{array}$$

The integral term is used for dynamic adjustment of the threshold to accommodate changes in the anomaly score.

To better understand the definition of the anomaly indicator, we introduce the following supplemental formulas:

Adjusted Mean:


$$\mu_{S}^{\prime }=\mu_{S}+\int_{-\infty }^{\mu_{S}}f(x)\;dx$$

Here, $\mu_{S}^{\prime }$ is the adjusted mean, which better captures the tail characteristics of the anomaly scores.

Adjusted Standard Deviation:


$$\sigma_{S}^{\prime }=\sigma_{S}+\int_{0}^{1}h(S_{i},t^{\prime })\;dt^{\prime }$$

$\sigma_{S}^{\prime }$ is the adjusted standard deviation to increase the model’s sensitivity to anomalies.

Final Threshold Calculation:


$$\tau^{\prime }=\mu_{S}^{\prime }+\delta \cdot \sigma_{S}^{\prime }$$

The final threshold is calculated using the adjusted mean and standard deviation.

Anomaly Condition:

The final condition to determine whether node *i* is an anomaly is given by:


$$I_{i}=\{\begin{array}{cc} 1,\; & \text{if }S_{i}>\tau^{\prime }\\ 0,\; & \text{otherwise} \end{array}$$

Through this process, we derive the final expression for the anomaly indicator $I_{i}$, which includes several adjustment terms to improve the accuracy and robustness of anomaly detection.

## References

[1] Zheng X, Cai J, Zhang G. Stock trend prediction based on ARIMA-LightGBM hybrid model. In: 2022 3rd Information Communication Technologies Conference (ICTC). 2022. p. 227–31. doi: 10.1109/ictc55111.2022.9778304

[2] Xu Y, Zhang Y, Liu P, Zhang Q, Zuo Y. GAN-enhanced nonlinear fusion model for stock price prediction. Int J Comput Intell Syst. 2024:17(1);12.

[3] Dong C, Liu J, Lu Y, Zhang L. Stock value prediction based on merging SARIMA model and Monte Carlo model. In: Proceedings of the 2022 13th International Conference on E-Education, E-Business, E-Management and E-Learning (IC4E). ACM; 2022.

[4] Wang J, Liu D, Jin L, Sun Q, Xue Z. A PCA-IGRU model for stock price prediction. J Internet Technol. 2023;24(3):621–9.

[5] Liu W, Gui Z, Jiang G, Tang L, Zhou L, Leng W. Stock volatility prediction based on transformer model using mixed-frequency data. Lect Notes Comput Sci. 2024;14332:74–88.

[6] Lv P, Shu Y, Xu J, Wu Q. Modal decomposition-based hybrid model for stock index prediction. Exp Syst Appl. 2022;202:117252. doi: 10.1016/j.eswa.2022.117252 PMC8871263 35205442

[7] Zhang Q, Zhang Y, Bao F, Liu Y, Zhang C, Liu P. Incorporating stock prices and text for stock movement prediction based on information fusion. Eng Appl Artif Intell. 2024;127:107377. doi: 10.1016/j.engappai.2023.107377

[8] Muhammad T, Aftab AB, Ibrahim M, Ahsan MdM, Muhu MM, Khan SI, et al. Transformer-based deep learning model for stock price prediction: a case study on Bangladesh stock market. Int J Comp Intel Appl. 2023;22(03). doi: 10.1142/s146902682350013x

[9] Zhang J, Ye L, Lai Y. Stock price prediction using CNN-BiLSTM-attention model. Mathematics. 2023; 11(9):1985.

[10] Yang Z, Zhao T, Wang S, Li X. MDF-DMC: a stock prediction model combining multi-view stock data features with dynamic market correlation information. Exp Syst Appl. 2024;238:122134. doi: 10.1016/j.eswa.2023.122134

[11] Agrawal M, Kumar Shukla P, Nair R, Nayyar A, Masud M. Stock prediction based on technical indicators using deep learning model. Comput Mater Continua 2022;70(1):287–304. doi: 10.32604/cmc.2022.014637

[12] Biju AKVN, Thomas AS, Thasneem J. Examining the research taxonomy of artificial intelligence, deep learning & machine learning in the financial sphere—a bibliometric analysis. Quality Quantity 2024;58(1):849–78. doi: 10.1007/s11135-023-01426-1 PMC10153784 37359968

[13] Sahu SK, Mokhade A, Bokde ND. An overview of machine learning, deep learning, and reinforcement learning-based techniques in quantitative finance: recent progress and challenges. Appl Sci 2023;13(3):1956. doi: 10.3390/app13031956

[14] Dong X, Dang B, Zang H, Li S, Ma D. The prediction trend of enterprise financial risk based on machine learning ARIMA model. J Theory Pract Eng Sci. 2024;4(1):65–71.

[15] AbdelKawy R, Abdelmoez WM, Shoukry A. A synchronous deep reinforcement learning model for automated multi-stock trading. Prog Artif Intell 2021;10(1):83–97. doi: 10.1007/s13748-020-00225-z

[16] Wang J, Zhang S, Xiao Y, Song R. A review on graph neural network methods in financial applications. arXiv preprint 2021

[17] Wang B, Dong Y, Yao J, Qin H, Wang J. Exploring anomaly detection and risk assessment in financial markets using deep neural networks. IJIRCST 2024;12(4):92–8. doi: 10.55524/ijircst.2024.12.4.15

[18] Yu W, Jin D, Zhao F, Zhang X. Towards pilot’s situation awareness enhancement: a framework of adaptive interaction system and its realization. ISA Trans. 2023;132:109–19. doi: 10.1016/j.isatra.2022.12.005 36567190

[19] Zhang L, Wang R, Li Z, Li J, Ge Y, Wa S, et al. Time-series neural network: a high-accuracy time-series forecasting method based on Kernel filter and time attention. Information 2023;14(9):500. doi: 10.3390/info14090500

[20] Elrefaei I, Khatib N. Faculty of Electrical Engineering and Communication. Theses Publication; 2022.

[21] Dahal KR, Gupta A, Pokhrel NR. Predicting the direction of NEPSE index movement with news headlines using machine learning. Econometrics 2024;12(2):16. doi: 10.3390/econometrics12020016

[22] Soltani H, Taleb J, Ben Hamadou F, Boujelbène-Abbes M. Using machine learning to forecast clean energy, commodities, green bonds and ESG index prices: how important is financial stress?. EMJB. 2024. doi: 10.1108/emjb-12-2023-0341

[23] Chen J, Wu J. The prediction of Chongqing’s GDP based on the LASSO method and chaotic whale group algorithm-back propagation neural network-ARIMA model. Sci Rep 2023;13(1):15002. doi: 10.1038/s41598-023-42258-z 37696872 PMC10495363

[24] Shaban WM, Ashraf E, Slama AE. SMP-DL: a novel stock market prediction approach based on deep learning for effective trend forecasting. Neural Comput Appl 2023;36(4):1849–73. doi: 10.1007/s00521-023-09179-4

[25] Jeribi F, Martin R, Mittal R, Jari H, Alhazmi A, Malik V. A deep learning based expert framework for portfolio prediction and forecasting. IEEE Access. 2024. doi: 10.1109/ACCESS.2024.1234567

[26] Setiawan H, Bhaduri M. Spotting the stock and crypto markets’ rings of fire: measuring change proximities among spillover dependencies within inter and intra-market asset classes. Appl Netw Sci 2023;8(1):61. doi: 10.1007/s41109-023-00589-w

[27] Baltakienė M, Kanniainen J, Baltakys K. Identification of information networks in stock markets. J Econ Dyn Control. 2021;131:104217. doi: 10.1016/j.jedc.2021.104217

[28] Bao W, Cao Y, Yang Y, Che H, Huang J, Wen S. Data-driven stock forecasting models based on neural networks: a review. Inf Fusion. 2025;113:102616. doi: 10.1016/j.inffus.2024.102616

